# Capture of Longitudinal Change in Real‐Life Walking in Cerebellar Ataxia Increases Patient Relevance and Effect Size

**DOI:** 10.1002/mds.30230

**Published:** 2025-05-21

**Authors:** Jens Seemann, Theresa Beyme, Natalie John, Florian Harmuth, Martin Giese, Ludger Schöls, Dagmar Timmann, Matthis Synofzik, Winfried Ilg

**Affiliations:** ^1^ Section Computational Sensomotorics Hertie Institute for Clinical Brain Research Tübingen Germany; ^2^ Centre for Integrative Neuroscience (CIN) Tübingen Germany; ^3^ Division Translational Genomics of Neurodegenerative Diseases, Hertie‐Institute for Clinical Brain Research and Center for Neurology University of Tübingen Tübingen Germany; ^4^ Institute of Medical Genetics and Applied Genomics, University Hospital Tübingen University of Tübingen Tübingen Germany; ^5^ German Center for Neurodegenerative Diseases (DZNE) Tübingen Germany; ^6^ Department of Neurodegeneration Hertie Institute for Clinical Brain Research and Centre of Neurology Tübingen Germany; ^7^ Department of Neurology and Center for Translational and Behavioral Neurosciences (C‐TNBS), Essen University Hospital University of Duisburg‐Essen Essen Germany

**Keywords:** biomarker, cerebellar ataxia, digital health, real‐life walking, wearable sensors

## Abstract

**Background:**

With disease‐modifying drugs for degenerative ataxias on the horizon, ecologically valid measures of gait performance that can detect patient‐relevant changes in trial‐like time frames are highly warranted.

**Objectives:**

In this 2‐year longitudinal study, we aimed to unravel ataxic gait measures sensitive to longitudinal changes in patients' real lives using wearable sensors.

**Methods:**

We assessed longitudinal gait changes of 26 participants with degenerative cerebellar disease (Scale for the Assessment and Rating of Ataxia [SARA]: 9.4 ± 4.1) at baseline, 1‐year, and 2‐year follow‐up using three body‐worn inertial sensors in two conditions: (1) laboratory‐based walking (LBW); and (2) real‐life walking (RLW). In RLW, a context‐sensitive analysis was performed by selecting comparable walking bouts according to macroscopic gait characteristics. Gait analysis focused on measures of spatio‐temporal variability, particularly stride length variability, lateral step deviation, and a compound measure of spatial variability (SPCmp).

**Results:**

Gait variability measures showed high test–retest reliability in both walking conditions (intraclass correlation coefficient [ICC], ≥0.82). Cross‐sectional analyses revealed high correlations of gait measures with ataxia severity (SARA, effect size ρ ≥ 0.75); and with patients' subjective balance confidence (Activity‐specific Balance Confidence scale [ABC]: ρ ≥ 0.71).

Although SARA showed longitudinal changes only after 2 years, the gait measure *SPCmp* revealed changes after 1 year with high effect size (*r*_prb_ = 0.80). Sample size estimation for the gait measure SPCmp showed a required cohort size of n = 42 participants (n = 38; spinocerebellar ataxias [SCA]_1/2/3_ subgroup) to detect a 50% reduction in progression at 1 year with a hypothetical intervention, compared to n = 147 for SARA at 2 years.

**Conclusions:**

Because of their ecological validity and larger effect sizes, real‐life gait characteristics represent promising performance measures as outcomes for future treatment trials. © 2025 The Author(s). *Movement Disorders* published by Wiley Periodicals LLC on behalf of International Parkinson and Movement Disorder Society.

Gait measures constitute promising candidates for performance outcome measures in upcoming therapeutic intervention studies in ataxias,^1^, ^2^ because gait disturbances often present as the first signs of degenerative cerebellar disease (DCD)^3^, ^4^, ^5^ and represent one of the most patient‐relevant disabling features throughout the disease course.^6^, ^7^ It has been shown in laboratory‐based assessments by different capture technologies that measures of spatio‐temporal variability allow to characterize ataxic gait^8^, ^9^, ^10^, ^11^, ^12^, ^13^, ^14^ (for reviews, see Buckley et al,^15^ Milne et al,^16^ Ilg et al,^17^ and Cabaraux et al^18^) with high sensitivity to ataxia severity in cross‐sectional and first longitudinal studies.^19^, ^20^

With progress in wearable sensor technology enabling gait recordings in patients' real life, it was hypothesized that those real‐life gait measures could be potentially even more sensitive to disease‐specific signatures of ataxic gait impairment compared to clinical and lab settings, because of the complexity and challenges of the environments,^21^, ^22^, ^23^, ^24^, ^25^ but also because of the larger amount of available walking bouts.^26^ In a first cross‐sectional study on real‐life gait in degenerative cerebellar ataxia, we have shown that ataxic‐sensitive gait measures allow not only to capture the gait variability inherent in ataxic gait in real life, but also to demonstrate high sensitivity to small cross‐sectional differences in disease severity, with higher effect sizes in real‐life walking (RLW) compared to clinical gait assessment.^27^ However, to serve as ecologically valid patient‐focused progression and therapy response outcomes, these gait measures have to prove (1) their sensitivity to individual longitudinal change in a short period realistic for intervention trials;^1^, ^28^ and (2) their meaningfulness by anchoring with patient‐reported outcomes, as required by the United States Food and Drug Administration (FDA).^29^

Noteworthy, to compare patients' real‐life gait behavior at two measurement time points, the influence of context and environment on gait measures have to be considered. These contextual and environmental factors have been shown to have a significant impact on macroscopic (ie, behavioral, as opposed to microscopic, ie, spatio‐temporal)^30^, ^31^ gait characteristics, such as speed, length of walking bouts, and number of turns.^25^, ^32^, ^33^, ^34^ These gait characteristics will differ for indoor (eg, in a small apartment) versus outdoor walking, and in turn will influence several gait measures,^30^, ^35^ as it has been shown in healthy participants and for different patient populations (Parkinson's disease, multiple sclerosis, cerebral palsy).^35^, ^36^, ^37^ This holds for general performance measures like mean gait speed and even more for variability measures.^36^, ^37^, ^38^ Analysis of shorter walking bouts for indoor walking—compared to longer outdoor bouts—inherently provides increased variability for healthy participants and patients.^37^, ^39^

Therefore, if one compares measures of a patient's gait variability at two time points 1 year apart, increased variability could be caused not by increased balance disturbances, but rather by mere differences in the context and environmental factors of the recorded gait behavior. Therefore, to identify disease progression or treatment‐induced changes in longitudinal analyses of real‐life gait behavior, it is necessary to consider these contextual and environmental factors, particularly their influence on macroscopic gait characteristics.

Here, we performed a longitudinal analysis of baseline, 1‐ and 2‐year follow‐up gait recordings in lab‐based gait assessment and patients' real life. Matching of longitudinal walking bouts (baseline and follow‐up) was performed according to macroscopic characteristics of walking behavior, namely the bout length and number of turns. We hypothesized that gait measures capturing longitudinal change in patients' real life could be (1) more sensitive to progression in short, trial‐like time‐frames (eg, 1 year) compared to lab‐based gait assessments and clinical rating scales; and (2) more patient‐relevant in terms of correlation with patient‐reported outcomes of balance confidence in important activities of everyday living. This would be key for future treatment trials, as the targeted primary outcome is usually a slowing of disease progression in a limited study period, ideally capturable within 1 year, and by outcomes reflecting patient relevance, as emphasized by the FDA.^29^

## Patients and Methods

### Participants and Clinical Outcome Assessments

#### Study Participants

Twenty‐six participants at an early‐to‐moderate ataxic or pre‐ataxic stage of DCD (age: 48 ± 9.5 years) were recruited from the Ataxia Clinics of the University Hospitals Tübingen and Essen. They consisted of 21 participants in the ataxic (ATX) stage of DCD as defined by a Scale for the Assessment and Rating of Ataxia (SARA) score of ≥3 (group ATX; SARA: 9.4 ± 3.2), and five participants with repeat‐expansions in spinocerebellar ataxia SCA2, SCA3, or SCA6 at the pre‐ataxic (PRE) stage of DCD (SARA<3) (group PRE: SARA: 1.6 ± 0.65).^40^ A total of 18 of 26 DCD participants carried a repeat expansion in SCA1, 2, or 3 (SCA_1/2/3_ subgroup). All main analyses were additionally performed in this subgroup, because these fast‐progressing polyglutamine (polyQ) SCA types are a promising target in many upcoming intervention trials.^1^, ^2^ Details of patient characteristics are shown in Table 1.

**TABLE 1:** Patient characteristics at baseline assessment.

Patient	Diagnosis	SARA^BL^	SARA_p&g_^BL^	Stage^BL^
PRE1	SCA6	2.5	0	0
PRE2	SCA2^Conv^	2	0	0
PRE3	SCA3	1	0	0
PRE4	SCA3	1	0	0
PRE5	SCA3	1.5	0	0
ATX1	SCA6	3	0	0
ATX2	SCA3	8.5	4	2
ATX3	ADCD	4.5	2	1
ATX4	ADCK3	8.5	3	2
ATX5	SCA3	13	6	2
ATX6	SCA2	4.5	1	1
ATX7	SCA14	10	4	2
ATX8	ADCK3	10	5	2
ATX9	PNPLA6	9.5	4	2
ATX10	SCA1	12	5	2
ATX11	SCA2	12.5	5	2
ATX12	SCA3	13	5	2
ATX13	SCA3	9	4	2
ATX14	SCA6	8.5	2	1
ATX15	SCA1	17.5	6	2
ATX16	SCA1	10.5	4	2
ATX17	SCA3	6.5	3	2
ATX18	SCA1	9	3	2
ATX19	SCA3	7.5	3	2
ATX20	SCA3	9.5	5	2
ATX21	SCA3	10	4	2
Subgroup PRE	#5 age Ø45.2 ± 10.1 BMI Ø23.8 ± 2.5	SARA Ø1.6 ± 0.7	T2EDO Ø: −3.8 [−11,3]	
Subgroup ATX	#21 age Ø49.8 ± 9.1 BMI Ø24.4 ± 3.7	SARA Ø9.4 ± 3.2		
Group DCD	#26 age Ø48.9 ± 9.5 BMI Ø24.3 ± 3.5	SARA Ø7.9 ± 4.2		
Controls HC	#34 age Ø44.08 ± 14.8 BMI Ø25.3 ± 4.5			

Clinical ataxia severity was determined by the SARA.^40^ Cerebellar patients include pre‐ataxic (PRE: SARA<3) and ataxic participants (ATX: SARA≥3). Participant PRE2 converted after 1 year to the ataxic stage (Conv). SARA scores and FARS ataxia staging^41^ (stage) are shown at BL. T2EDO was defined for the PRE subgroup as the difference between current age and estimated age at onset,^42^ with estimated disease onset calculated based on the individual's CAG repeats, as described in Tezenas du Montcel et al.^43^ Negative values denote estimated disease onset in the future, positive values denote estimated disease onset in the past. The following diagnosis denotes the gene underlying the respective ataxia type: ADCK3 (=ARCA 2, autosomal‐recessive cerebellar ataxia type 2); *PNPLA6*.

Abbreviations: SARA, Scale for the Assessment and Rating of Ataxia; BL, baseline; SARA_p&g_ Scale for the Assessment and Rating of Ataxia posture and gate; PRE, pre‐ataxic; SCA, autosomal‐dominant spinocerebellar ataxia of defined genetic type; ATX, ataxic participants; ADCD, autosomal dominant ataxia of still undefined genetic cause; BMI, body mass index; T2EDO, time to disease onset; HC, healthy controls.

Patients were included based on the following inclusion criteria: (1) degenerative cerebellar ataxia in the absence of any signs of secondary central nervous system disease; (2) age between 18 and 75 years; and (3) ability to walk without walking aids. Exclusion criteria were: severe visual or hearing impairment, cognitive impairment that limits the understanding of instructions or the performance of the gait tasks in the laboratory and real life, or orthopedic limitations (eg, severe arthrosis or lower limb fractures or hip/knee replacements) that functionally affect gait. In addition, we recruited 34 healthy controls (HC) (age: 44.08 ± 14.78 years). Healthy participants had no history of any neurological or psychiatric disease, no family history of neurodegenerative disease, and did not show any neurological signs on clinical examination. Participants were analyzed cross‐sectionally at baseline and, where available, longitudinally at 1‐year and 2‐year follow‐ups.

#### Clinical Outcome Assessments and Patient‐Reported Outcomes

The severity of ataxia was rated using the SARA.^40^ The three items rating gait and posture are grouped by the subscore SARA posture and gait (SARA_p&g_).^14^, ^44^ SARA assessments were performed by expert ataxia neurologists (M.S., L.S., and D.T.) To capture the impact of disease on daily living, DCD participants were asked to self‐report their balance confidence in activities important in daily living using the activity‐specific balance confidence scale (ABC).^45^

### Standard Protocol Approvals, Registration, and Patient Consent

The experimental procedure was approved by the local ethics committee (598/2011BO1; 303/2008BO2). All participants gave their informed consent before participation.

### Gait Conditions

Walking movements were recorded in two different conditions. (1) Lab‐based walking (LBW), where participants walked 60 m straight on a 30 m indoor floor (ie, including one turn) at their preferred self‐selected speed on a pre‐specified straight route in an institutional setting, supervised without any distractions. The turn and one stride before and after the turn were excluded from the analysis. (2) RLW, which is unconstrained walking during participants' usual individual everyday living, where participants were free to move how they wanted and were used to in their daily life, without supervision by any study personnel (total recording time: 4–6 hours within 2–3 days). Participants were instructed to wear the sensors inside and outside their homes and include at least a half‐hour walk. Participants were instructed to wear the sensors for consecutive recording sessions, each lasting a maximum of 2 hours. Participants documented their recorded walking movements in an activity protocol (for details see Supplement S1).

### Movement Recordings and Gait Measures

Three Opal inertial sensors (APDM, Portland, OR) were attached on both feet and posterior trunk at the level of L5 with elastic Velcro bands. Inertial sensor data was collected and wirelessly streamed to a laptop for the automatic generation of gait and balance metrics by Mobility Lab software (APDM). For the real‐life condition, data was logged on board of each OPAL sensor and downloaded after the session. Step events and spatio‐temporal measures were extracted using Mobility Lab (Version 2),^46^ which has been shown to deliver good‐to‐excellent accuracy and repeatability.^47^, ^48^

From the rich source of gait measures, we adopted a hypothesis‐driven approach, focusing on those measures that have been considered promising candidate gait measures in previous work. Recent longitudinal studies from our group in laboratory‐based gait analysis identified 1‐year longitudinal gait change in a SCA3^19^ and an early SCA2 cohort,^20^ revealing stride length variability (StrideL_CV_), lateral body sway, lateral step deviation (LatStepDev) and a compound measure of spatial step variability (SPCmp, combining StrideL_CV_ and LatStepDev)^27^ (see Supplementary S2) as most sensitive to longitudinal change in ataxia severity. In RLW, we showed that LatStepDev and SPCmp were sensitive to the cross‐sectional ataxia severity,^27^ and to short‐term treatment‐related improvements in SCA27B.^49^

In addition, we included speed as an indicator of functional mobility and the variability of the lateral angle of the foot during the stance phase (ToeOutAngle_SD_, inspired by results in Shah et al^12^). The lateral sway was determined by the coronal range of motion measured by the lumbar sensor (CorRoM_SD_). StrideL_CV_ was determined using the coefficient of variation (CV) = σ/μ, normalizing the standard deviation (SD) with the mean value.

### Selected Walking Bouts and Matching of RLW Behavior

The analysis focused on walking bouts >15 strides, as gait variability measures in short walking bouts are often estimated inaccurately.^26^ The first and last two strides of each bout were removed to reduce the effects of gait initiation and termination on the variability measures.^50^ A walking bout is here defined as a sequence of strides that is not interrupted by a turn or a complete halt of at least two median gait cycle durations of the individual. Finally, bouts that showed a jump in gait speed of at least 0.5 m/s between two strides were discarded.

### Matching of RLW Behavior Based on Macroscopic Gait Characteristics

Because of the described influence of contextual and environmental factors on macroscopic gait characteristics,^30^, ^31^ we used a linear regression model to confirm that gait variability measures like LatStepDev are influenced by bout length and the number of turns (number of turns, 60 seconds before and after the bout, see Supplement S3).

Second, we introduced a matching procedure for identifying for each subject comparable walking bouts in longitudinal assessments. This matching procedure compares walking bouts and identifies matching pairs by optimizing a cost function with the two macroscopic gait descriptors: bout length and number of turns.

**FIG. 1:**
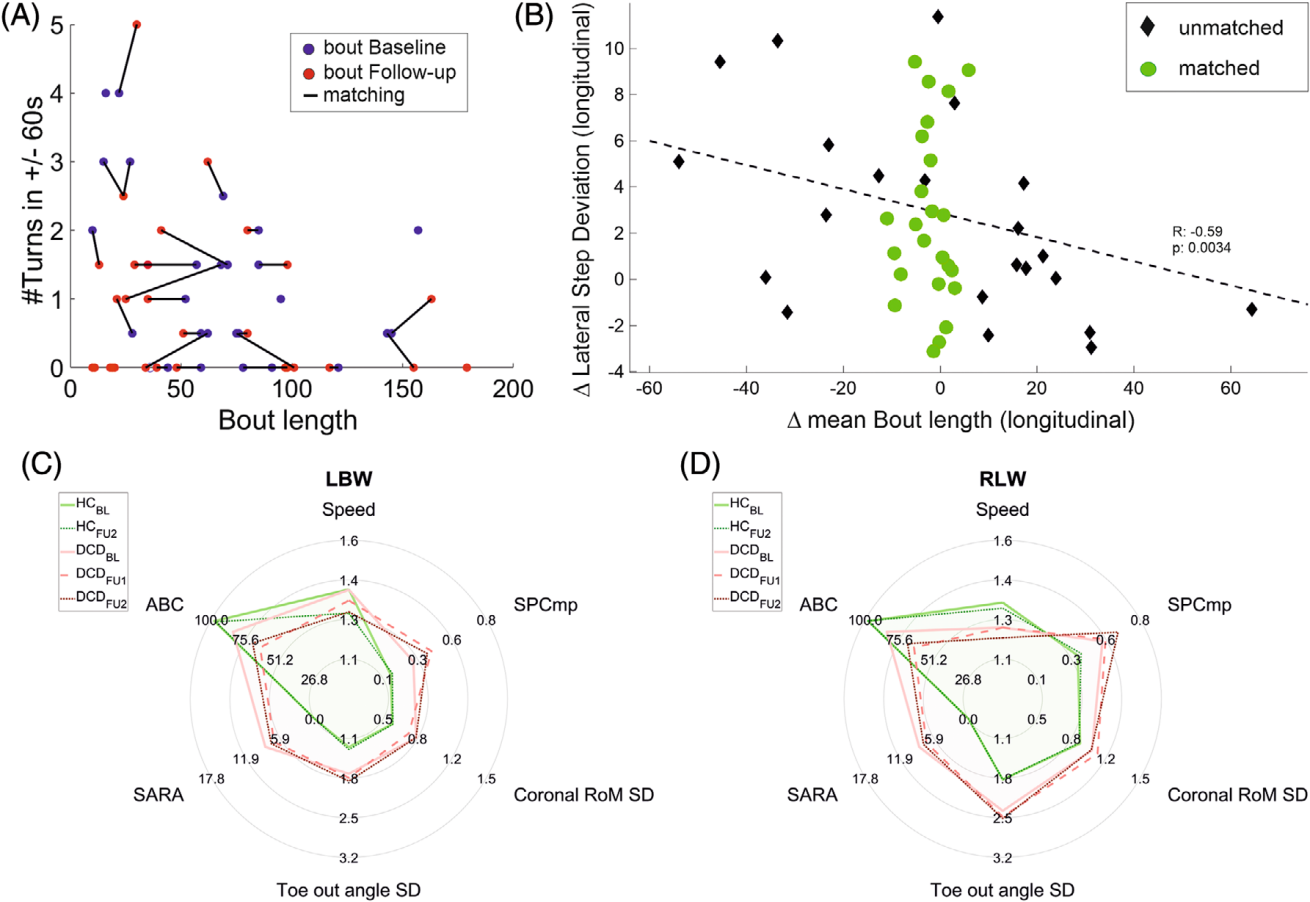
(**A**) Illustration of the mapping procedure of corresponding real‐life walking (RLW) trials at baseline (blue) and the 1‐year follow‐up (red). (**B**) Relationship between longitudinal differences (∆ = 1‐year follow‐up – baseline) differences in mean bout length and lateral step deviation for the degenerative cerebellar disease (DCD) participants (non‐matched: red; matched: blue). In the non‐matched condition, there is a significant correlation between the longitudinal difference in lateral step deviation and the difference in mean bout length (**r** = 0.59, *P* = 0.0034**). (**C**,**D**) Radar plots illustrating cross‐sectional and longitudinal differences on Scale for the Assessment and Rating of Ataxia, Activity‐specific Balance Confidence scale, and four gait parameters for the gait conditions laboratory‐based walking LBW (**C**) and RLW (**D**): speed, spatial variability compound measure (SPCmp), coronal range of motion variability (CorRoM_SD_), and foot angle variability (ToeOutAngle_SD_). Cross‐sectional differences can be seen by comparison of healthy controls at baseline (HC_BL_) and the DCD group at baseline (DCD_BL_). Given are average values for each group. Longitudinal progression can be seen comparing DCD participants at baseline (DCD_BL_) and follow‐up (FU) assessments (DCD_FU1_, DCD_FU2_) as well as for healthy controls (HC_BL_ and HC_FU2_). [Color figure can be viewed at wileyonlinelibrary.com]

For matching, an Euclidean distance matrix D was created for each subject using bout length and number of turns for each baseline bout to each follow‐up bout (see Fig. 1A). To weight both criteria equally, the data were previously standardized per subject (centered and normalized to one SD of the participants' baseline measurement). Using the distance matrix D for each subject as a cost matrix, a linear assignment problem was solved to obtain a 1:1 matching of similar bouts for baseline and follow‐up. A cost for non‐assignment of 0.5 was set, to obtain bouts that were as similar as possible, but at the same time to avoid losing any participants for the analysis. Therefore, for each subject, only bouts with similar macroscopic gait descriptors are compared. Mismatched bouts are not included in the analysis, reducing purely contextual longitudinal differences.

To determine test–retest reliability in everyday life, we used the matching procedure described above. Here, the two baseline measurement days with the most bouts were selected and matched. For the LBW, we divided the 60 m task into two 30 m segments (before and after the turn) and calculated the split‐half reliability (see Statistics). The test–retest reliability of gait measures was calculated using the intraclass correlation (ICC) (2, 1).^51^ ICC values <0.5, between 0.5 and 0.75, between 0.75 and 0.9, and >0.90 were considered as poor, moderate, good, and excellent reliability, respectively.^51^ Based on the ICC the minimum detectable change (MDC) was calculated, which is critical in determining whether a change can be reliably detected or is lost in the measurement noise.^52^

$${\mathrm{MDC}}_{90}=1.65\times {\mathrm{SD}}_{\text{baseline}}\times \left(\surd 2\left[1-\mathrm{ICC}\right]\right)$$

With 1.65 is the z‐score of 90% level of confidence. Please note that we used a conservative method for calculating the MDC, which is suitable for individual measures. For groups, the original term can be divided by $\sqrt{n}$, with (n = group size).^53^, ^54^

### Statistics

Between‐group differences (DCD vs. HC) were determined using the non‐parametric Mann–Whitney *U* test with effect sizes calculated by Cliff's Δ.^55^ Repeated measurement analyses were performed for longitudinal analyses using the non‐parametric Friedman‐Test to determine within‐group differences between assessments. When the Friedman‐Test yielded an effect (*P* < 0.1), post hoc analysis was performed using a Wilcoxon‐signed‐rank‐test for pairwise comparisons. Effect sizes r_prb_ for the repeated measurements analyses were determined by matched‐pairs rank biserial correlation.^56^

We report three significance levels: (1) uncorrected *P < 0.05; (2) Bonferroni‐corrected for multiple comparisons **P < 0.05/n = 6: number of analyzed features; and (3) ***P < 0.001. Spearman's ρ was used to examine the correlation between movement measures and SARA, between gait measures for different walking conditions, and between gait measures for matched and unmatched walking bouts. Effect sizes ρ were classified as ρ: 0.1 small effect, 0.3 medium effect, 0.5 large effect, 0.7 very large effect.^57^ Statistical analysis was performed using MATLAB (The MathWorks, version R2024A, Natick, MA). Based on the effect sizes of longitudinal change of the gait measures and the SARA, a sample size estimation was performed using G*power 3.1^58^ to determine the required cohort size for detecting a 50% reduction of progression by a hypothetical intervention.

## Results

Real‐life gait data was collected from 26 DCD participants at baseline (253 ± 206 minutes recorded within 2.1 ± 1.3 days), from 23 DCD at the 1‐year follow‐up after 380 ± 52 days (follow‐up 1 [FU1], 227 ± 147 minutes recorded within 2.2 ± 1.1 days) and from 22 DCD at the 2‐year follow‐up after 816 ± 106 days (follow‐up 2 [FU2], 326 ± 234 minutes recorded within 2.5 ± 1.1 days).

From the collected RLW gait data, we extracted 92,117 valid strides in 2854 bouts (length: 47.2 ± 32.6 strides). After the described matching procedure, 53,178 strides in 894 bouts (length: 58.6 ± 63.9 strides) were analyzed. For comparison, the amount of gait data extracted from LBW was in total 1021 strides in 52 bouts (length: 24.7 ± 4.2 strides).

### Sensitivity of Gait Measures to Ataxia Severity: Cross‐Sectional Results

Cross‐sectional analyses revealed group differences between DCD versus HC for several examined gait variability measures in both walking conditions, constrained LBW (StrideL_CV_: *P* = 0.002**; LatStepDev: *P* = <0.001***; SPCmp: *P* = <0.001***) and RLW (LatStepDev: *P* = <0.001***; SPCmp: *P* < 0.001***) (see Table 2, Fig. 1C,D). High sensitivity of gait measures StrideL_CV_, LatStepDev, and SPCmp to cross‐sectional ataxia severity was indicated by correlations with the SARA and the SARA_p&g_ subscore, with large effect size in both conditions ρ > 0.75. In addition, gait measures revealed high correlations with the patient‐reported balance confidence in activities of daily living, assessed by the ABC (StrideL_CV_, LatStepDev, SPCmp: *P* < 0.001***) (Table 2). Effect sizes were higher for RLW than LBW (*SPCmp*: ρ = −0.81 RLW vs. ρ = −0.71 LBW).

**TABLE 2:** Cross‐sectional analyses: Between‐group differences of HC and DCD participants for clinician reported outcomes, patient‐reported outcomes, and gait measures in the LBW and RLW conditions at baseline assessment.

	Measure	Group difference DCD vs. HC		Correlations DCD				
				SARA		ABC		
		*P*	δ	ρ	*P*	ρ	*P*	ICC
Clinician‐reported outcomes	SARA	<0.001***	0.99	–	–	0.76	0.001**	–
	SARA_p&g_	<0.001***	0.75	–	–	0.73	0.002**	–
Patient‐reported outcome	ABC	<0.001***	0.70	–	–	–	–	–
Gait–LBW	Speed	0.67	0.06	0.03	0.88	−0.22	0.45	0.94
	StrideL_CV_	0.002**	0.47	0.79	<0.001***	−0.55	0.04*	0.73
	LatStepDev	<0.001***	0.64	0.81	<0.001***	−0.71	0.005**	0.91
	SPCmp	<0.001***	0.60	0.81	<0.001***	−0.71	0.005**	0.83
	CorRoM_SD_	<0.001**	0.54	0.74	<0.001***	−0.72	0.004**	0.75
	ToeOutAng_SD_	<0.001**	0.62	0.64	<0.001***	−0.70	0.006*	0.49
Gait–RLW	Speed	0.19	0.2	−0.47	0.02*	0.05	0.86	0.83
	StrideL_CV_	0.06	0.29	0.75	<0.001***	−0.35	0.23	0.84
	LatStepDev	<0.001***	0.61	0.77	<0.001***	−0.81	<0.001***	0.82
	SPCmp	<0.001***	0.54	0.78	<0.001***	−0.81	<0.001***	0.85
	CorRoM_SD_	0.13	0.23	0.59	0.002**	−0.62	0.02*	0.84
	ToeOutAng_SD_	0.008**	0.41	0.59	0.002**	−0.27	0.36	0.79

δ Denotes the effect sizes determined by Cliff's δ. Correlations between gait measures and clinician‐reported ataxia severity (SARA, SARA_p&g_) and patient‐reported outcomes (ABC) are given for the DCD group. Effect sizes of correlations are given using Spearman's ρ. Test–retest reliability is analyzed by determining the ICC (see Patients and Methods).

*Indicates significant differences between groups (* ≡ *P* < 0.05, ** ≡ *P* < 0.0083 Bonferroni‐corrected, *** ≡ *P* < 0.001).

Abbreviations: HC, healthy controls; DCD, degenerative cerebellar disease; LBW, lab‐based waling; RLW, real‐life walking; ICC, intraclass correlation coefficient; SARA, Scale for the Assessment and Rating of Ataxia; ABC, Activity‐specific Balance Confidence scale; SARA_p&g_, Scale for the Assessment and Rating of Ataxia posture and gait; m, mean; SD, standard deviation; StrideL_CV_, stride length variability; LatStepDev, lateral step deviation; SPCmp, compound measure of spatial variability; CorRoM_SD_, coronal range of motion measured by the lumbar sensor; ToeOutAng_SD_, foot angle variability.

### Gait Measures for Matched Walking Bouts Show Good‐to‐Excellent Test–Retest Reliability

To identify suitable macroscopic gait characteristics for matching walking bouts, linear regression analysis showed significant contributions of both, bout length and number of turns in explaining the variability for walking bouts of HC (see Supplement S3). Performing the presented matching procedure on the real‐life baseline assessment (see Patient and Methods), revealed good to excellent test–retest reliability (ICC) for several gait measures like LatStepDev, StrideL_CV_, and SPCmp (ICC (1, 2) ≥0.82) (see Table 2).

### Sensitivity of Gait Measures to Longitudinal Change

Longitudinal analyses revealed for the SARA significant changes only in the second follow‐up (see Table 3). The patient‐reported outcome measure of balance confidence (ABC score) as well as a few laboratory‐based gait measures revealed significant changes after 1 year with modest effect sizes (ABC: FU1: *P* = 0.03*, *r*_prb_ = −0.52; speed: FU1: *P* = 0.04*, *r*_prb_ = −0.47; CorRoM_SD_: FU1: *P* = 0.03*, *r*_prb_ = 0.51), whereby the latter grow substantially after 2 years (eg, speed: FU2: *P* = 0.0005***, *r*_prb_ = −0.84; StrideL_CV_: FU2: *P* = 0.005**, *r*_prb_ = 0.68) (Table 3).

**TABLE 3:** Longitudinal within‐subject comparison for the group of DCD.

Group		Friedman test		Baseline	1‐year follow‐up				2‐year follow‐up				
DCD	Measure	χ^2^	*P*	M ± SD	M ± SD	*P*	*r*_prb_	ESS_50%_	M ± SD	*P*	*r*_prb_	ESS_50%_	MDC_90_
Clinician‐reported outcomes	SARA	1.42	0.49	8.0 ± 4.5	8.1 ± 4.5	0.66	0.12	–	9.1 ± 5.5	0.02*	0.50	147	–
	SARA_p&g_	3.04	0.21	3.1 ± 2.1	3.0 ± 2.1	0.79	0.17	–	3.5 ± 2.5	0.03*	0.53	179	–
Patient‐ reported outcome	ABC	1.87	0.39	75.6 ± 20.5	67.7 ± 23.4	0.03*	−0.52	73	68.7 ± 31.2	0.12	−0.51	–	–
Gait–LBW	Speed	12.0	0.001^+^	1.39 ± 0.14	1.35 ± 0.12	0.04*	−0.47	84	1.26 ± 0.19	0.0005***	−0.84	66	0.019 ^**<FU1+2**^
	StrideL_CV_	1.9	0.38	0.02 ± 0.007	0.023 ± 0.016	0.30	0.24	–	0.029 ± 0.02	0.005**	0.68	67	0.0065^**<FU2**^
	LatStepDev	3.4	0.17	0.032 ± 0.011	0.034 ± 0.013	0.44	0.18	–	0.036 ± 0.015	0.04*	0.47	51	0.003^**<FU2**^
	SPCmp	6.0	0.04^+^	0.38 ± 0.21	0.41 ± 0.21	0.27	0.26	–	0.47 ± 0.23	0.04*	0.48	39	0.04 ^**<FU2**^
	CorRoM_SD_	6.5	0.03^+^	0.87 ± 0.34	0.99 ± 0.34	0.03*	0.51	83	0.91 ± 0.33	0.7	0.11	–	0.2
	ToeOutAng_SD_	0.6	0.7	1.81 ± 0.5	1.79 ± 0.61	0.54	−0.14	–	2.00 ± 0.72	0.13	0.36	–	0.54
Gait–RLW	Speed	4.1	0.12	1.23 ± 0.14	1.17 ± 0.13	0.09	−0.39	–	1.15 ± 0.2	0.009**	−0.63	50	0.048 ^**<FU1+2**^
	StrideL_CV_	5.5	0.06^+^	0.034 ± 0.012	0.042 ± 0.016	0.0047**	0.67	41	0.048 ± 0.03	0.04*	0.49	69	0.0025 ^**<FU1+2**^
	LatStepDev	5.1	0.07^+^	0.044 ± 0.009	0.047 ± 0.01	0.05	0.44	45	0.048 ± 0.008	0.006**	0.66	43	0.0021 ^**<FU1+2**^
	SPCmp	10.9	0.004^+^	0.58 ± 0.17	0.67 ± 0.2	0.0007***	0.80	42	0.72 ± 0.31	0.001**	0.78	54	0.02 ^**<FU1+2**^
	CorRoM_SD_	7.6	0.02^+^	1.0 ± 0.21	1.09 ± 0.2	0.006**	0.65	43	1.07 ± 0.24	0.18	0.32	–	0.13
	ToeOutAng_SD_	3.37	0.18	2.35 ± 0.57	2.56 ± 0.59	0.04*	0.47	62	2.68 ± 0.9	0.12	0.37	–	0.22^**<FU2**^

Shown are results of clinician‐reported ataxia ratings (SARA score and SARA_p&g_ subscore^44^) as well as of gait measures in clinical assessment LBW and RLW for baseline, 1‐year, and 2‐year follow‐up assessments. Friedman test determined within‐group longitudinal differences (^+^ *P* < 0.1). Post hoc test *P*‐values determined by Wilcoxon signed‐rank test for both follow‐up assessments relative to baseline. Effect sizes r_prb_ determined by matched‐pairs rank‐biserial correlation.^56^ Shown are analyses for the whole group of DCD, both consisting of pre‐ataxic and ataxic participants. For significant longitudinal changes, we determined the sample size estimate for a cohort required to detect a 50% reduction in progression ESS_50%_. MCD_90_ values denote the smallest reliable detectable change (90% confidence interval). Superscripts indicate whether the MDC is smaller than the change between baseline and 1‐year follow‐up (‘^<FU1’^) or/and between baseline and 2‐year follow‐up.

*Indicate significant differences between groups (* ≡ *P* < 0.05, ** ≡ *P* < 0.0083 Bonferroni‐corrected, *** ≡ *P* < 0.001).

Abbreviations: DCD, degenerative cerebellar disease; MDC, minimum detectable change; M, mean; SD, standard deviation; SARA, Scale for the Assessment and Rating of Ataxia; SARA_p&g_, Scale for the Assessment and Rating of Ataxia posture and gait; ABC, Activity‐specific Balance Confidence scale; LBW, lab‐based walking; StrideL_CV_, stride length variability; LatStepDev, lateral step deviation; SPCmp, compound measure of spatial variability; CorRoM_SD_, coronal range of motion measured by the lumbar sensor; ToeOutAng_SD_, foot angle variability; RLW, real‐life walking.

**FIG. 2:**
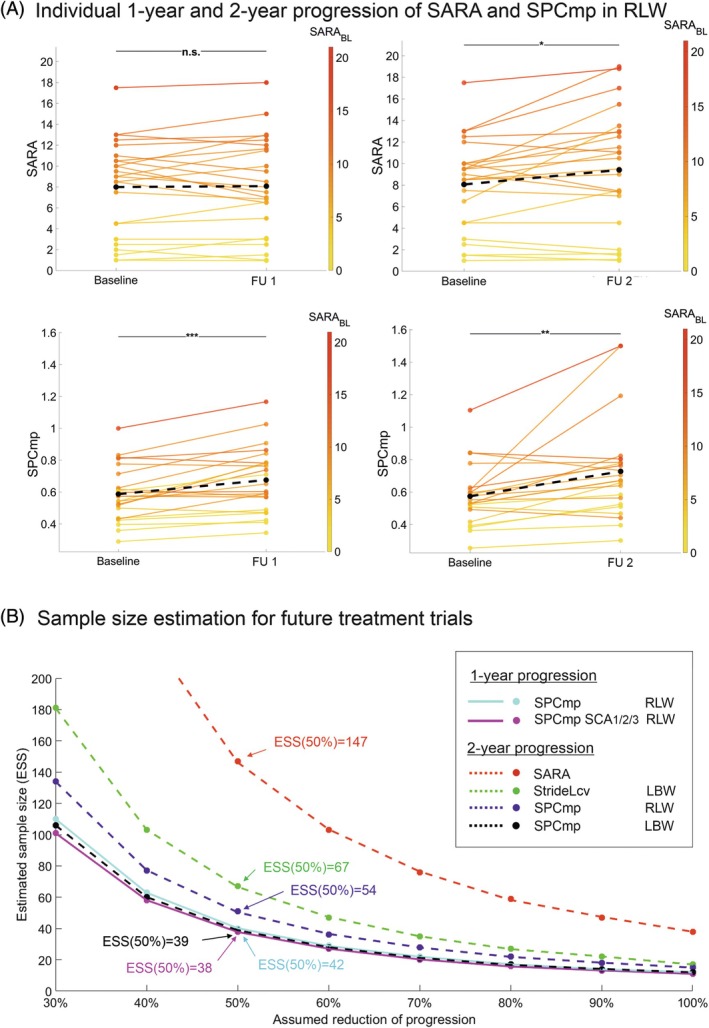
(**A**) Within‐subject changes between baseline and follow‐up assessments for the group of degenerative cerebellar disease (DCD) participants. Shown are longitudinal differences in Scale for the Assessment and Rating of Ataxia (SARA) (upper panel) and the spatial variability compound measure (SPCmp) (lower panels). For both measures, the left panel shows the change between baseline and 1‐year follow‐up (FU1), and the right panel the change between baseline and 2‐year follow‐up (FU2). In all panels, SARA scores of individual cerebellar participants are color‐coded. Black dotted line = mean change across all participants. Stars indicate significant differences between time points (* ≡ *P* < 0.05, ** ≡ *P* < 0.0083 Bonferroni‐corrected, *** ≡ *P* < 0.001). (**B**) Sample size estimates were made for future treatment trials that showed different levels of progression reduction for the various outcome measures: SARA as well as the gait measures SPCmp and stride length variability (StrideLcv) in the laboratory (LBW) and real life (RLW). The estimated number of participants per study arm is plotted against the hypothesized therapeutic effect on reducing 1‐year progression or 2‐year progression in DCD patients or the SCA_1/2/3_ subgroup (SCA_1/2/3_), respectively. Solid lines indicate 1‐year estimates, and dashed lines indicate 2‐year estimates. [Color figure can be viewed at wileyonlinelibrary.com]

In contrast, in condition RLW, several gait measures—in particular StrideL_CV_ and SPCmp—revealed significant changes with high effect sizes already after 1 year (StrideL_CV_: FU1: *P* = 0.0047**, *r*_prb_ = 0.67; SPCmp: FU1: *P* = 0.0007***, *r*_prb_ = 0.80). The robustness of these results is supported by the second follow‐up (SPCmp: FU2: *P* = 0.016*, *r*_prb_ = 0.77) (Figure 2A).

Moreover, these longitudinal changes after 1 year for the gait measures StrideL_CV_ and SPCmp can be observed and validated in the subgroup SCA_1/2/3_ with higher effect size (FU1: n = 18; FU2: n = 18) (SPCmp: FU1: *P* = 0.0023**, *r*_prb_ = 0.86; FU2: *P* = 0.0067**, *r*_prb_ = 0.76) (Supplement S4). For both the DCD group and the SCA_1/2/3_ subgroup, these annual changes were larger than the MDC (see Tables 3 and Supplement‐S4) (eg, SPCmp: ∆_BL vs. FU1_ = 0.12 > MDC_90_ = 0.02). In the HC group, longitudinal changes in the RLW condition were observed in gait speed, but not in the ataxic‐specific measures of spatio‐temporal variability (see Fig. 1C,D).

Based on observed effect sizes in the DCD population, sample size estimation for SPCmp showed for the RLW condition a required cohort size of n = 42 for detecting a 50% reduction of progression after 1 year by a hypothetical intervention (90% power and one‐sided 5% type I error) in comparison to n = 147 for the SARA (Fig. 2B, Table 3). For the SCA_1/2/3_ subgroup, because of the larger effect size, the required cohort size was even smaller (n = 38). In the LBW condition, the required cohort size for SPCmp was n = 39 after 2 years (Fig. 2B, Table 3).

Compared to the matched walking trials in RLW, analysis of the non‐matched walking trials (bout length > 15) showed significant longitudinal differences, yet with smaller effect sizes (SPCmp: FU1: *P* = 0.0089*, *r*_prb_ = 0.62; FU2: *P* = 0.01*, *r*_prb_ = 0.58) (Supplement‐S5). Moreover, the analysis of the non‐matched walking bouts revealed a correlation between longitudinal differences in variability measures like StrideL_CV_ and longitudinal differences in mean bout length (*r* = 0.59, *P* = 0.0034**), potentially influencing the observed changes. Such correlations were not observed in the matched analysis (Figure 1B).

## Discussion

This study aimed to test the hypothesis that the progression of ataxic‐related gait impairments can be reliably and sensitively captured in real‐life walking by analysis of walking bouts, which are matched according to macroscopic gait characteristics reflecting environmental factors. We showed that real‐life gait measures can capture longitudinal change within short trial‐like time frames like 1 year with high effect size, therefore, outperforming in their sensitivity both clinician‐reported outcomes like the SARA and lab‐based gait measures. These results indicate an increased sensitivity and ecological validity for real‐life gait measures as promising performance outcomes in upcoming treatment trials.

### Increased Spatio‐Temporal Gait Variability as a Consistent Feature of Ataxic Gait in Laboratory‐Based Assessments

Our findings in the constrained walking condition LBW confirm the results of previous studies by our group and others using different motion capture technologies.^8^, ^9^, ^12^, ^13^, ^19^ These studies showed that spatio‐temporal variability measures serve as reliable and valid measures for cerebellar ataxia in constrained walking conditions, and correlate with gait and posture ataxia severity and with patients' subjective balance confidence in important activities of daily living (ABC score).

### Measures of Ataxic Gait in Real Life: Cross‐Sectional Sensitivity to Ataxia Severity

Although real‐life gait is inherently more variable in both HC and cerebellar patients,^39^ several of our gait variability measures (eg, StrideL_CV_, LatStepDev, and SPCmp), allow to capture the ataxia‐specific gait variability in straight walking episodes of real life.^27^ The compound measure SPCmp—integrating variability in the anterior–posterior and in the mediolateral dimension—hereby seems to benefit from capturing individual differences in gait variability.^27^

Consistent with previous studies in lab‐based gait assessments, cross‐sectional sensitivity of real‐life gait measures (eg, StrideL_CV_, LatStepDev, and SPCmp) for ataxia severity was shown by the high correlation with clinical severity of ataxia (*P* < 0.002**) (see Table 2).

Even more meaningful for patient relevance and ecological validity, however, is the high correlation between gait measures and patients' subjective balance confidence in daily living, as assessed by the ABC score. The high effect sizes—even higher for real‐life than lab‐based gait assessments (SPCmp: RLW: ρ = 0.81, LBW: ρ = 0.71)—emphasize the relevance of ataxia‐related gait measures to patients' everyday life—which is key to FDA‐conform patient‐focused outcome and drug development.^29^

### Matching Walking Bouts According to Bout Length and Number of Turns

The influence of contextual and environmental factors on gait measures during real‐life walking is currently under intense investigation in various movement disorders.^25^, ^34^, ^36^, ^38^ To date, matching or selection procedures based on contextual factors or macroscopic gait characteristics have been used exclusively in cross‐sectional studies, for example, to compare a group of patients with HC in activity monitoring and real‐life walking, to differentiate patient subgroups,^25^ or to compare patients' real‐life walking behavior with clinical gait assessments.^38^, ^59^ Moreover, in most approaches, the selection of walking sessions was based on bout length alone.

In contrast, the focus of our study was to investigate a matching approach as a novel strategy for longitudinal change analysis, allowing to identify comparable walking bouts at baseline and follow‐up visits. As turns are an important part of real‐life walking behavior, and typically differ between indoor and outdoor walking,^32^ we included the number of turns and the bout length in our macroscopic characterization of walking behavior to identify comparable walking bouts. The number of turns contributes to the macroscopic walking characteristics in explaining step variability, as observed by linear regression analysis (Supplement S3). For the longitudinal analyses, the matching procedure ensures that observed effects are not predominantly because of differences in bout length, which is important to avoid false positive results regarding the progression of the gait variability measures. Using the proposed matching procedure, we showed good‐to‐excellent test–retest reliability for real‐life recordings on different days.

In summary, this approach allows comparison of real‐life gait assessments without requiring participants to perform similar gait behavior in all home assessments or capturing home data over a long period (where contextual differences can be assumed to average out). At the same time, it highlights the need for other longitudinal gait studies to control for changes in macroscopic walking characteristics like bout length and the number of turns, as potential confounders.

### Gait Measures in RLW Capture Longitudinal Change with Higher Effect Size

Using these matching procedures, and consistent with the cross‐sectional results, measures of spatio‐temporal variability in real‐life walking (and in particular the compound measure SPCmp) show high responsiveness to change at 1 year. Their effect size in longitudinal sensitivity to change outperforms the clinician‐reported outcome (SARA) and laboratory gait measures.

Importantly, the high test–retest reliability under real‐life conditions resulted in a longitudinal change that was higher than the MDC (see Table 3), which is a critical requirement for reliable detection in clinical trials.^60^

The subgroup SCA_1/2/3_ showed higher effect sizes and, consequently smaller sample size estimates. This finding is consistent with previous studies showing faster progression in SCA 1, 2, 3^61^ compared to a cross‐genotype DCD population, which includes slower progressing DCD types (eg, SCA6^62^ and non‐PolyQ SCAs).^63^

The reduction in sample size inferred by these digital performance outcomes could be decisive for the feasibility of a treatment trial: whereas trials with, for example, 147 SCA participants per trial arm (as required for SARA as outcome for 2 years) are almost impossible, 38 SCA participants (as required for the gait measure SPCmp in SCA_1/2/3_ for 1 year) (Figure 2B) are well feasible.

The potential of real‐life gait assessments is also supported by the comparison of the results presented here with a previous study on laboratory‐based gait assessment in an SCA2 population, which found significant changes in laboratory‐based gait measures.^20^ Although the smaller number of SCA patients and the addition of slower progressing DCD types in the current study resulted in a non‐significant change in laboratory‐based scores after 1‐year, real‐life scores showed a significant change.

### Limitations

Overall, the present study aimed to explore and longitudinally validate digital performance measures in real‐life ataxic gait with a cross‐genotype cohort, as this approach allows validation across DCDs. This approach was based on the assumption that our measures would capture functional impairment generically across DCDs, given that they qualitatively affect the same ataxia‐related functions. This assumption is corroborated by our previous work demonstrating validity of ataxia‐specific gait measures across various DCDs^20^, ^49^, ^64^ and by the comparable longitudinal results for the DCD cohort and SCA_1/2/3_ subgroup observed in the current study. However, given that different DCD genotypes gradually differ in their specific progression rates, the sensitivity of digital gait measures to detect longitudinal changes formally remains to be demonstrated within specific genotypes and pre‐ataxic populations only. Furthermore, additional work should investigate longer—completely uninstructed—real‐life assessments to (1) include other important components of walking behavior (eg, turning,^23^ gait initiation,^65^ and termination^66^) in addition to straight walking episodes; and (2) quantify daily fluctuations in patients' walking behavior.

Multicenter studies need to further investigate the potential of real‐life gait assessments regarding their increased sensitivity and relevance,^24^, ^67^ but also consider multicenter challenges such as the necessity for patient compliance, the impact of walking aids,^60^ and the more complex data analysis compared to lab‐based gait assessments.

## Conclusion

This study unraveled methods and measures that allow quantifying longitudinal changes in real‐life ataxic gait in early to moderate disease stages with high effect sizes and high correlations with patient‐reported outcomes of daily living. This provides promising ecologically valid, patient‐centered outcome measures for natural history and treatment trials in degenerative cerebellar ataxias. In addition to the higher effect sizes gained from real‐life assessments, these measures allow for the objective quantification of patients' real‐life gait performance—instead of clinical assessment in partly artificial settings, for example, by gait tasks as part of clinical scores or under lab conditions, which serve as surrogate parameters at best.

In conclusion, measures of real‐life gait performance add ecological validity, and therefore, help to inform upcoming treatment trials in cerebellar ataxias and FDA‐compatible development of patient‐focused outcomes and approval of novel treatments.^21^, ^28^

## Author Roles

(1) Research project: A. Conception, B. Organization, C. Execution; (2) Statistical Analysis: A. Design, B. Execution, C. Review and Critique; (3) Manuscript Preparation: A. Writing of the First Draft, B. Review and Critique.

J.S.: 1A, 1B, 1C, 2B, 3A

T.B.: 1C, 3B

N.J.: 1C, 3B

F.H.: 1C, 3B

M.G.: 1B, 1C, 3B

L.S.: 1B, 1C, 3B

D.T.: 1B, 1C, 3B

M.S.: 1A, 2C, 3A

W.I: 1A, 1B, 1C, 2A, 2B, 3A

## Financial Disclosure

W.I. received consultancy honoraria by Ionis Pharmaceuticals, unrelated to the present work. J.S., T.B., N.J., F.H., M.G., and D.T. report no disclosures. L.S. served as advisor for Alexion, Novartis, and Vico. He participates as a principal investigator in clinical studies sponsored by Vigil Neuroscience (VGL101‐01.001; VGL101‐01.002), Vico Therapeutics (VO659‐CT01), PTC Therapeutics (PTC743‐NEU‐003‐FA), and Stealth BioTherapeutics (SPIMD‐301), all unrelated to the present work. M.S. has received consultancy honoraria from Ionis, UCB, Prevail, Orphazyme, Biogen, Servier, Reata, GenOrph, AviadoBio, Biohaven, Zevra, Lilly, and Solaxa, all unrelated to the present manuscript.

## Supporting information


**Data S1** Supporting Information.

## Data Availability

The data that support the findings of this study are available on request from the corresponding author. The data are not publicly available due to privacy or ethical restrictions.
