# Supplementary material for: Capture of Longitudinal Change in Real‐Life Walking in Cerebellar Ataxia Increases Patient Relevance and Effect Size
Source: Mov Disord. 2025 May 21;40(7):1343–55. doi: 10.1002/mds.30230 (PMC12273614; doi:10.1002/mds.30230)
Supplement: Supplementary file 1 — Data S1 Supporting Information. [file MDS-40-1343-s001.docx]

# Supplementary information

## Supplement S1- Capturing real-life movements - Instructions and usability

All participants in our study were trained by technical staff to apply the sensors for home use (RLW) after completion of the laboratory measurement (LBW). Furthermore, the wearable sensors were previously labelled with unique letters in the correct orientation for the user to prevent incorrect positioning of the sensors by the participant. To record the RLW, the participants were provided for at least 3 days with 3 Opal sensors, a docking station and power supply, step-by-step instructions on how to set up the sensors and document the recorded activity, and a mobile phone number of the technical staff to clarify any queries. The following instructions for RLW were given verbally and in text form:

"Please move naturally as part of your normal daily routine. Please do not shower, bathe or walk in the rain with the sensors on. For longer periods of rest with little or no activity, or before going to bed at night, simply remove the sensors and charge them using the docking station provided. We ask you to document your recorded activities in the activity log provided (approximately every 15 to 30 minutes). For technical reasons, it is advisable to return the sensors to the docking station after a maximum of 2 hours in order to save the data collected so far.”

The data was stored in logging mode on the internal memory of the sensors, before being sent by parcel post to the University Hospital of Tübingen. Thereafter, data was downloaded and analysed by technical staff.

Moreover, upon completion of the measurements, the participants were asked to fill out a questionnaire on the usability of the sensors. This questionnaire comprised three questions on a Likert scale ranging from 1 to 5 (1='does not apply at all', 2='rather not apply', 3='neutral', 4='rather apply' and 5='fully apply'):

1. 'Setting up the OPAL sensor system was technically unproblematic for me.' (sensor attachment)

2. 'Wearing the system did not have a negative impact on my everyday movements: I was no more unsteady walking or standing than without the system.' (no influence on gait)

3. 'I could imagine wearing the system for 12 hours during the day as part of a therapy study.' (potential long-time usage)

The evaluation of this questionnaire for healthy controls (n=34), unimpaired patients (FARS stage 0&1, n=9) and minimally impaired patients (FARS stage 2, n=17) showed a high usability of the OPAL system used already at the baseline measurement (see Figure S1, mean values from 4.5 to 5). Only one subject in stage 2 had problems putting on the system (1 for ‘sensor attachment’) and needed help from a family member.


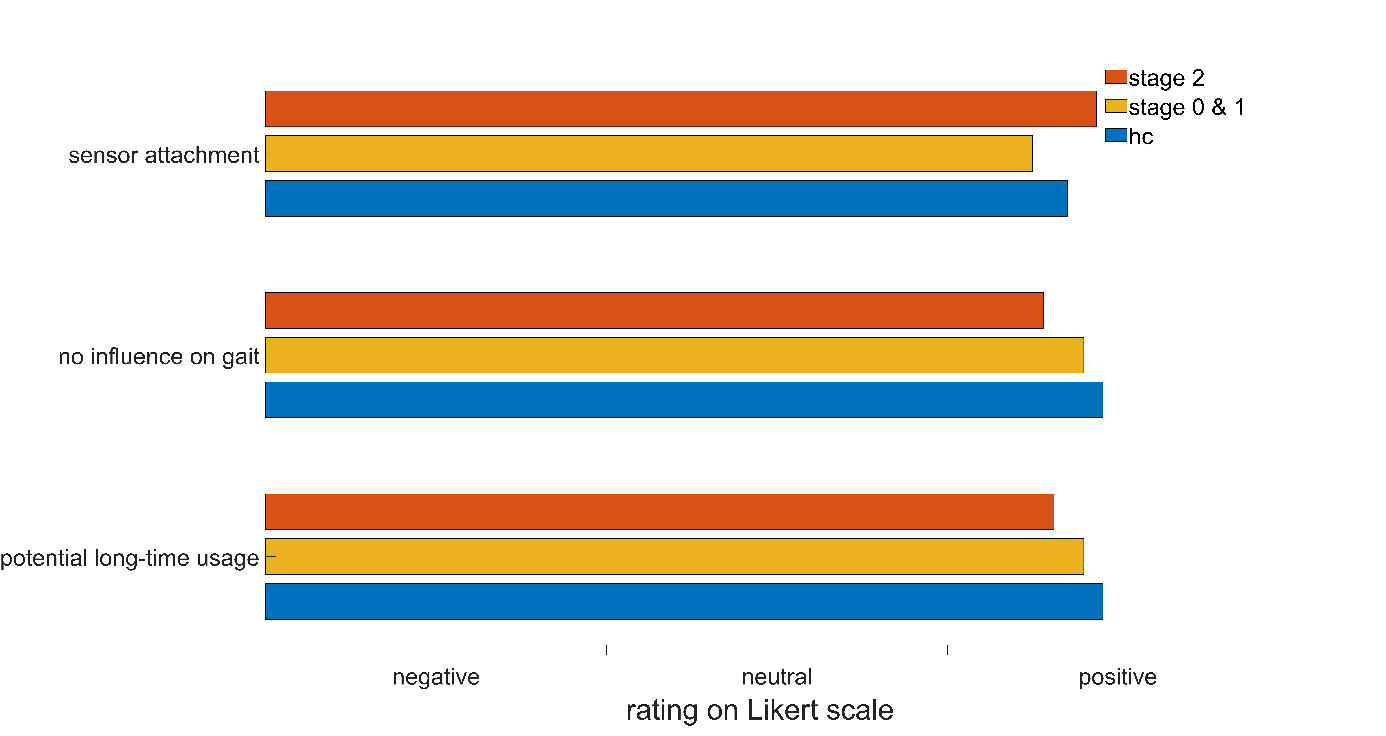


Figure S1 Usability of the OPAL system in everyday life, rated on a Likert scale of 1 (negative) to 5 (positive) in the cohorts of healthy controls (n=34), unimpaired patients (FARS stage 0&1, n=9) and mild-to-moderate impaired patients (FARS stage 2, n=17).

## Supplement S2- Details on gait measures

Lateral step deviation (*LatStepDev*): This gait measure was determined based on three consecutive walking steps, calculating the absolute amount of perpendicular deviation of the middle foot placement from the line connecting the first and the third step (Figure S2).

A two-strides sliding window was used over all strides to be analyzed and all absolute values were averaged over both legs. *LatStepDev* was normalized with stride length (% of stride length), thus providing a measure independent from stride length variability, which is suggested to be increased in real-life gait.

Coronal Range of Motion (*CorRoM*): The angular range of the thoracic spine in the coronal plane (roll).[^1^](#_ENREF_1)

Toe-out angle variability: Motivated by[^1^](#_ENREF_1), we examined an additional feature of variability, namely *toe-out angle_SD_*. Toe-out angle was determined as the lateral angle of the foot during the stance phase, relative to the forward motion of the gait cycle[^2^](#_ENREF_2). Positive angle is outward rotation.[^1^](#_ENREF_1) Increased toe-out angle has been shown to be associated with increased stride width, and variability in stride width is associated with dynamic postural instability[^1^](#_ENREF_1).


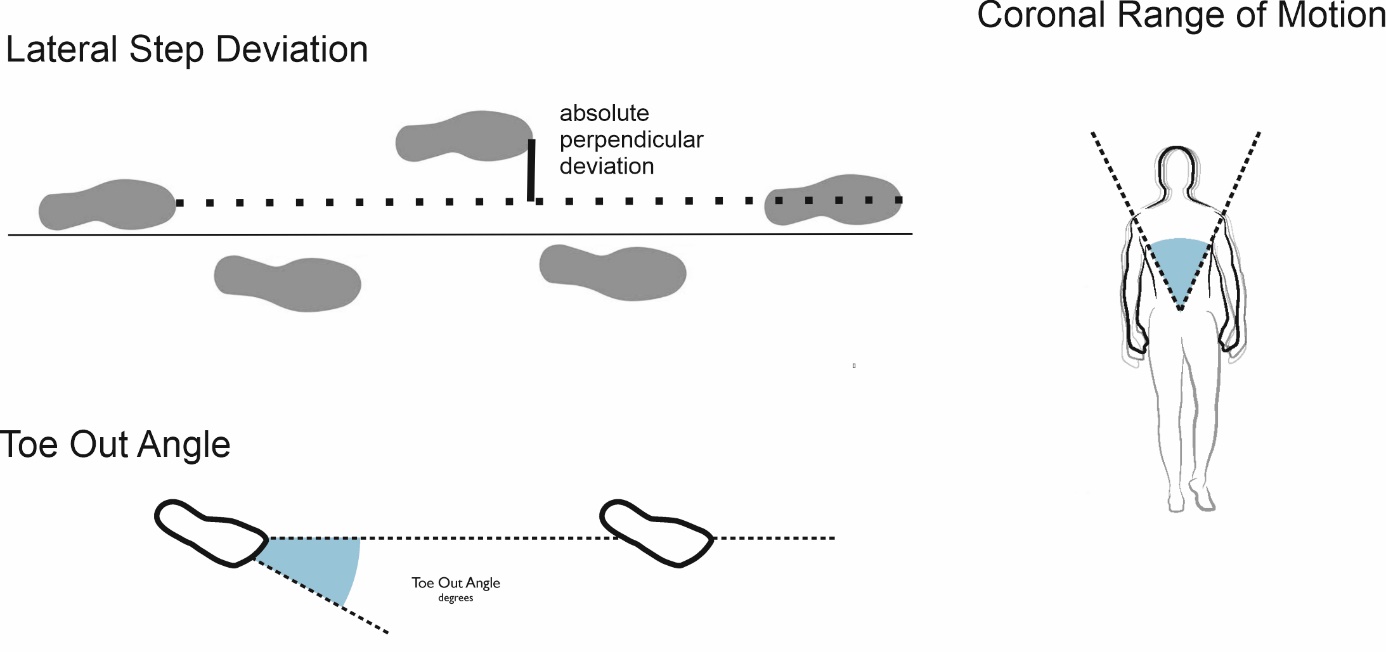


Figure S2 Illustration of the gait measures: Lateral step deviation, Toe Out Angle, and Coronal Range of Motion (Adopted from[^1^](#_ENREF_1)).

## Compound measure of spatial variability

The spatial step variability compound measure *SPCmp was determined in two steps: step one* determines for each of the two parameters (*StrideL_CV_*) and (*LatStepDev*) separately the relative value of an individual subject in comparison to the value range of all participants (resulting in values between [0-1], see Figure S2 A). In step 2, that measure out of these two measures was taken for final analysis where the individual’s result showed a larger abnormality (shown by a value nearer to 1), whereas the respective other measure was not entered into the further analysis, see Equation 1 and Figure S2-A. For the longitudinal analysis, the value range of all participants is determined based on the baseline assessment. Therefore, values in the follow-up assessment can exceed the range [0-1].

$SPcmp {(DCD}_{i})=\max\left( \left( \frac{{StrideL}_{CV} {DCD}_{i}-\min_{DCD+HC} {StrideL}_{CV}}{\max_{DCD+HC} S{trideL}_{CV}-\min_{DCD+HC} {StrideL}_{CV}} \right),\left( \frac{LatStepDev {DCD}_{i}-\min_{DCD+HC} LatStepDev}{\max_{DCD+HC} LatStepDev-\min_{DCD+HC} LatStepDev} \right) \right)$ (Eq.1)


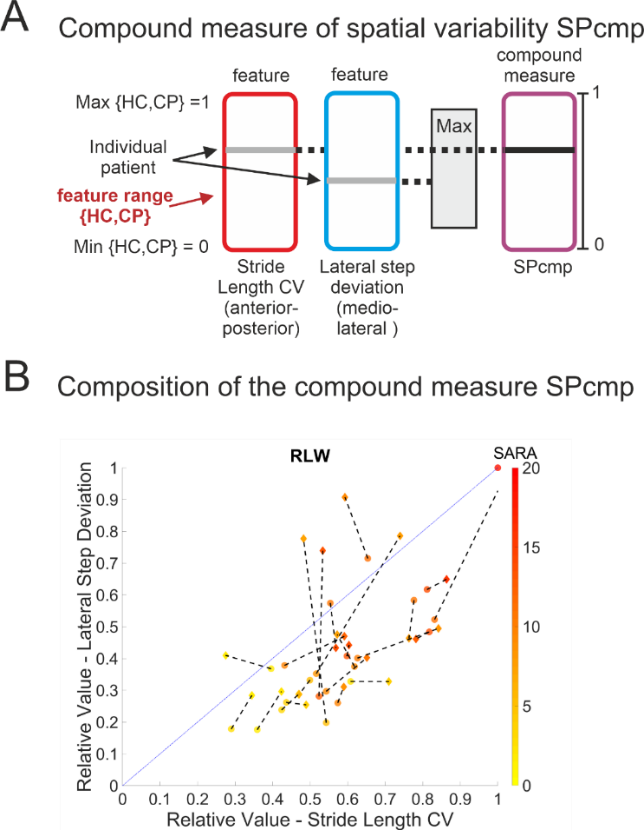


Figure S3 (A) Determination of the compound measure SPCmp. It is determined by the maximum of the relative values for the parameters StrideL_CV_ and LatStepDev. (B) Composition of the compound measure SPCmp for the walking conditions RLW in Baseline and the first follow-up assessment. Shown are the relative parameter values of each patient for the parameters StrideL_CV_ (x-axis) and LatStepDev (y-axis). The color-coding denotes the severity of gait and posture ataxia as determined by the SARA score. Dotted lines connect the baseline and the follow-up examination for the individual patients.

## Supplement S3 – Linear regression models of the matching procedure

We performed linear regression models to examine the influence of macroscopic gait characteristics on our variability measures.

Linear regression models:

The regression models examined the predictability of the gait measures lateral step deviation and stride length from different high-level gait descriptors like bout length, number of turns, SARA, or age of the participants. The regression was conducted on all ataxic and pre-ataxic participants in the baseline and follow-up condition, where each bout represented one data point.

Linear regression model:

LateralStepDeviation ~ 1 + Boutlength + #Turns + SARA + Age

Estimated Coefficients:

                    Estimate          SE         tStat       pValue

                   ___________    __________    _______    ___________

    (Intercept)     0.042623     0.0011047     38.583    4.1261e-276

    Boutlength     -4.0261e-05    3.2212e-06    -12.499     3.5455e-35

    #Turns          0.00035816    6.5527e-05     5.4659     4.8952e-08

    SARA            0.001407    5.1198e-05     27.481     4.025e-152

    Age            -3.327e-05    2.3935e-05      -1.39        0.16459

Root Mean Squared Error: 0.0154

R-squared: 0.223, Adjusted R-Squared: 0.222

F-statistic vs. constant model: 280, p-value = 7.86e-212

--------------------------------------------------------------------------

Linear regression model:

StrideLength_CV_ ~ 1 + Boutlength + #Turns + SARA + Age

Estimated Coefficients:

                    Estimate          SE         tStat       pValue

                   ___________    __________    _______    __________

    (Intercept)       0.055889     0.0037609     14.861     1.245e-48

  Boutlength        -7.3048e-05    1.0966e-05    -6.6611    3.0976e-11

    #Turns          0.0013206    0.00022308     5.9199    3.4995e-09

    SARA            0.002412     0.0001743     13.838    1.5078e-42

    Age            -0.00047262    8.1485e-05       -5.8    7.1564e-09

Root Mean Squared Error: 0.0524

R-squared: 0.0733, Adjusted R-Squared: 0.0723

F-statistic vs. constant model: 77, p-value = 6.05e-63

## Supplement S4 – Longitudinal within-subject comparison for the SCA_1/2/3_ subgroup

Table S4 Longitudinal within-subject comparison for the SCA_1/2/3_ subgroup. Shown are results of clinical ataxia ratings (SARA score and SARA_p&g_ posture&gait subscore^[3](#_ENREF_3)^) as well as of gait measures in clinical assessment (LBW) and in real life (RLW) for baseline, 1-year and 2-year follow-up assessments**.** Friedman test **determined within-group longitudinal differences (^+^, p<0.1).** Post-hoc test **p-**values determined by **Wilcoxon signed-rank test for both follow-up assessments relative to baseline**. Stars indicate significant differences between groups (*≡ p<0.05, **≡ p<0.0083 Bonferroni-corrected, ***≡ p<0.001). Effect sizes r_prb_ determined by matched-pairs rank-biserial correlation[^4^](#_ENREF_4). . MCD_90_ denotes the smallest reliable detectable change (90% confidence interval). The MDC column indicates whether this is smaller than the change between baseline and 1-year Follow-up (‘<FU1’) or/and between baseline and 2-year Follow-up (<FU1+2).

| GROUP |  | Friedman – test | |  | Baseline | |  | | | 1-year Follow-Up | | | |  | 2-year Follow-Up | | | | | |  | | MDC_90_ |
| --- | --- | --- | --- | --- | --- | --- | --- | --- | --- | --- | --- | --- | --- | --- | --- | --- | --- | --- | --- | --- | --- | --- | --- |
| SCA 1/2/3 | **Measure** | **χ2** | **p** |  | | **m ± sd** | | **m ± sd** | | | | **p** | **r_prb_** | | **m ± sd** | | **p** | | **r_prb_** | | |  |  |
| Clinician-reported outcomes | SARA | 0.5 | 0.77 |  | | 8.47±4.9 | | | 8.55±4.7 | | 0.59 | | 0.16 | | | 8.93±6.0 | | 0.18 | | 0.37 | | | - |
|  | SARA_p&g_ | 1.3 | 0.51 |  | | 3.3±2.2 | | | 3.31±2.23 | | 0.78 | | 0.22 | | | 3.4±2.6 | | 0.18 | | 0.49 | | | - |
| Patient- reported outcome | ABC | 2.5 | 0.28 |  | | 70.0±2.2 | | | 63.3±21.2 | | 0.04* | | -0.80 | | | 67.1±31.6 | | 0.29 | | -0.41 | | | - |
| Gait - | Speed | 8.5 | 0.01* |  | | 1.44±0.14 | | 1.37±0.13 | | | 0.01* | | 0.67 | | | 1.29±0.21 | | 0.0026^**^ | | 0.833 | | | 0.02 **^<FU1+2^** |
| LBW | StrideL_CV_ | 2.17 | 0.33 |  | | 0.019±0.007 | | 0.024±0.01 | | | 0.06 | | 0.52 | | | 0.029±0.024 | | 0.006^**^ | | 0.76 | | | 0.0064**^<FU2^** |
|  | LatStepDev | 3.2 | 0.20 |  | | 0.033±0.01 | | 0.034±0.013 | | | 0.3 | | 0.29 | | | 0.034±0.014 | | 0.25 | | 0.35 | | | 0.004 |
|  | SPCmp | 3.13 | 0.20 |  | | 0.384± 0.21 | | 0.42±0.21 | | | 0.055 | | 0.54 | | | 0.457±0.37 | | 0.03* | | 0.63 | | | 0.05 **^<FU2^** |
|  | CorRoM_SD_ | 6.2 | 0.04* |  | | 0.92±0.36 | | 1.06±0.33 | | | 0.06 | | 0.52 | | | 0.918±0.32 | | 0.84 | | 0.1 | | | 0.19 |
|  | ToeOutAng_SD_ | 0.4 | 0.79 |  | | 1.3±0.47 | | 1.84±0.6 | | | 0.87 | | 0.11 | | | 1.94±0.7 | | 0.48 | | 0.21 | | | 0.51**^<FU2^** |
| Gait - | Speed | 3.9 | 0.14 |  | | 1.24±0.16 | | 1.18±0.12 | | | 0.09 | | 0.47 | | | 1.16±0.23 | | 0.01* | | 0.7 | | | 0.04 **^<FU1+2^** |
| RLW | StrideL_CV_ | 11.9 | 0.0026** |  | | 0.035±0.01 | | 0.047±0.017 | | | 0.0008^***^ | | 0.95 | | | 0.0497±0.03 | | 0.04* | | 0.6 | | | 0.0025 **^<FU1+2^** |
|  | LatStepDev | 2.03 | 0.36 |  | | 0.045±0.01 | | 0.047±0.012 | | | 0.19 | | 0.36 | | | 0.048±0.009 | | 0.04* | | 0.58 | | | 0.0018**^<FU1+2^** |
|  | SPCmp | 9.4 | 0.008** |  | | 0.60±0.19 | | 0.706±0.22 | | | 0.0023** | | 0.86 | | | 0.71±0.30 | | 0.0067^**^ | | 0.76 | | | 0.02**^<FU1+2^** |
|  | CorRoM_SD_ | 7.9 | 0.019* |  | | 1.05±0.19 | | 1.14±0.18 | | | 0.02* | | 0.64 | | | 1.09±0.26 | | 0.45 | | 0.23 | | | 0.2 |
|  | ToeOutAng_SD_ | 4.3 | 0.12 |  | | 2.33±0.62 | | 2.62±0.6 | | | 0.02* | | 0.63 | | | 2.67±1.02 | | 0.56 | | 0.18 | | | 0.22**^<FU1+2^** |

## Supplement S5 – Longitudinal within-subject comparison in the non-matched condition

Table S5 Longitudinal within-subject comparison for the group of degenerative cerebellar disease (DCD in the **non-matched condition**. Shown are gait measures in real life (RLW) for baseline, 1-year, and 2-year follow-up assessments**.** Friedman test **determined within-group longitudinal differences (^+^, p<0.1).** Post-hoc test **p-**values determined by **Wilcoxon signed-rank test for both follow-up assessments relative to baseline**. Stars indicate significant differences between groups (*≡ p<0.05, **≡ p<0.0083 Bonferroni-corrected, ***≡ p<0.001). Effect sizes r_prb_ determined by matched-pairs rank-biserial correlation[^4^](#_ENREF_4).

| non-matched |  | Friedman | |  | Baseline |  | 1-year Follow-Up | | | | |  | | | 2-year Follow-Up | | | |
| --- | --- | --- | --- | --- | --- | --- | --- | --- | --- | --- | --- | --- | --- | --- | --- | --- | --- | --- |
|  | **Measure** | **χ2** | **p** |  | **m ± sd** | **m ± sd** | | | **p** | | **r_prb_** | | | **m ± sd** | | **p** | **r_prb_** | |
| Gait - | Speed | 2.1 | 0.33 |  | 1.22±0.14 | 1.19±0.13 | | 0.33 | | 0.23 | | | 1.17±0.21 | | | 0.02^*^ | | 0.54 |
| RLW | StrideL_CV_ | 8.4 | 0.01^+^ |  | 0.036±0.013 | 0.04±0.015 | | 0.01^*^ | | 0.59 | | | 0.047±0.032 | | | 0.01^*^ | | 0.58 |
|  | LatStepDev | 10.8 | 0.006^+^ |  | 0.044±0.01 | 0.046±0.010 | | 0.07 | | 0.42 | | | 0.047±0.009 | | | 0.10 | | 0.39 |
|  | SPCmp | 14.9 | 0.0005^+^ |  | 0.57±0.16 | 0.63±0.2 | | 0.0089^**^ | | 0.62 | | | 0.71±0.40 | | | 0.01^*^ | | 0.58 |
|  | CorRoM_SD_ | 6.8 | 0.03^+^ |  | 1.01±0.19 | 1.08±0.19 | | 0.01^*^ | | 0.60 | | | 1.05±0.21 | | | 0.32 | | 0.24 |
|  | ToeOutAngle_SD_ | 8.2 | 0.01^+^ |  | 2.35±0.6 | 2.57±0.58 | | 0.0081^**^ | | 0.63 | | | 2.61±0.73 | | | 0.03^*^ | | 0.52 |

## Supplement S6 – Correlations of gait measures between lab-based walking and real-life walking

Table S6 Correlations between gait measures in the LBW and RLW conditions are shown for the DCD group at baseline. Effect sizes of correlations are given using Spearman’s ρ. (*≡ p<0.05, **≡ p<0.0083 Bonferroni-corrected, ***≡ p<0.001).

|  |  |  |  |
| --- | --- | --- | --- |
|  |  | **Corr _LBW, RLW_** | |
| Measure |  | ρ | **p** |
|  |  |  |  |
| Speed |  | 0.7 | 0.006^**^ |
| StrideL_CV_ |  | 0.81 | <0.001^***^ |
| LatStepDev |  | 0.74 | 0.002^**^ |
| SPCmp |  | 0.74 | 0.002^**^ |
| CorRoM_SD_ |  | 0.74 | 0.002^**^ |
| ToeOutAng_SD_ |  | 0.23 | 0.40 |

1. Shah VV, Rodriguez-Labrada R, Horak FB, et al. Gait Variability in Spinocerebellar Ataxia Assessed Using Wearable Inertial Sensors. Mov Disord 2021;36:2922-2931.

2. APDM. Whitepaper Mobility Lab [online]. Accessed 10.10.2022.

3. Lawerman TF, Brandsma R, Verbeek RJ, et al. Construct Validity and Reliability of the SARA Gait and Posture Sub-scale in Early Onset Ataxia. Front Hum Neurosci 2017;11:605.

4. Kerby DS. The Simple Difference Formula: An Approach to Teaching Nonparametric Correlation. Comprehensive Psychology 2014;3:11.IT.13.11.
